# Complete wedge resection for duodenal gastrointestinal stromal tumour: A case series of three patients

**DOI:** 10.1016/j.ijscr.2021.106674

**Published:** 2021-12-09

**Authors:** Tomoaki Ito, Tomoyuki Kushida, Mutsumi Sakurada, Kenichiro Tanaka, Koichi Sato, Hiroshi Maekawa

**Affiliations:** a Department of Surgery, Juntendo University Shizuoka Hospital, Juntendo University School of Medicine, Shizuoka 410-2295, Japan

**Keywords:** CT, computed tomography, EUS, endoscopic ultrasonography, GIST, gastrointestinal stromal tumour, HPF, high power field, IVC, inferior vena cava, LECS, laparoscopy endoscopy cooperative surgery, SMT, submucosal tumour, Gastrointestinal stromal tumour, Gastrointestinal stromal tumours, Duodenum, Wedge resection, Case series

## Abstract

**Introduction:**

Duodenal gastrointestinal stromal tumours (GIST) are rare. Therefore, difficulties are experienced when selecting the appropriate surgical procedure in patients with duodenal GISTs. This report presents the cases of three patients with duodenal GISTs who underwent wedge resection. This report would help surgeons identify clinical features and surgical procedures in patients with duodenal GISTs.

**Presentation of case:**

Three patients were diagnosed with duodenal submucosal tumours. The first patient presented with melena, the second with postoperative anaemia, and the third with an incidental finding of a large abdominal tumour after presenting with ischaemic colitis. All tumours arose in the 2nd portion of the duodenum and measured 3.5, 3, and 9.2 cm, respectively. Wedge resection of the duodenum was performed in all patients. In patients one and two, simple closure of duodenal wall was performed after wedge resection. In patient three, side-to-side anastomosis with the jejunum was performed because a large area of the wall was removed using the wedge resection technique. Pancreatoduodenectomy was avoided in all patients. Recurrence was not noted in any patient.

**Discussion:**

Since GISTs are not generally associated with lymph node metastasis, local resection with negative margins is sufficient to surgically manage patients with GISTs.

**Conclusion:**

Our results indicated the effectiveness of performing wedge resection for duodenal GISTs not in close proximity to the ampulla of Vater. Moreover, less invasive procedures should be adopted in patients with duodenal GISTs.

## Highlights

• GIST are derived from the intestinal cells of Cajal in the GIT and mesentery.
• The occurrence of duodenal GIST is rare.
• Complete wedge resection was performed for GIST at the 2nd portion of the duodenum.
• Wedge resection is a feasible and safe surgical method for duodenal GISTs.

## 1 Introduction

Gastrointestinal stromal tumours (GISTs) are mesenchymal tumours originating from the intestinal cells of Cajal in the gastrointestinal tract and mesentery [1]. Most GISTs primarily affect the stomach (60%), followed by the small intestine (35%), rectum, oesophagus, omentum, and mesentery [2]. Duodenal GISTs account for approximately 12–18% of all small intestinal GISTs [3] and are relatively rare.

Herein, we report the cases of three patients with GISTs in the 2nd portion of the duodenum who underwent wedge resection. Pancreatoduodenectomy was avoided in all patients. Although wedge resection of the duodenum for GISTs has been selected as the common procedure, difficulties are often encountered in selecting appropriate surgical procedures for these tumours. This report would help all surgeons learn clinical features and the surgical procedure for duodenal GISTs. This study was performed in line with the PROCESS 2020 Guideline [4].

## 2 Methods

This is a retrospective case series. We encountered three cases in our Department of Surgery between 2014 and 2018. The research registry number in accordance with the Declaration of Helsinki is researchregistry7409.

All patients' data and images were anonymised. No precautions were taken before intervention.

All patients were treated by a senior associate professor. The operation was performed under general anaesthesia with all three patients in the supine position. Anastomosis or closure of the duodenum was performed by a hand-sewn technique using absorbent silk threads.

We performed a clinical 5-year follow-up using computed tomography (CT) scanning in all patients at our hospital.

## 3 Case presentation

### 3.1 Case report 1

**Fig. 1:**
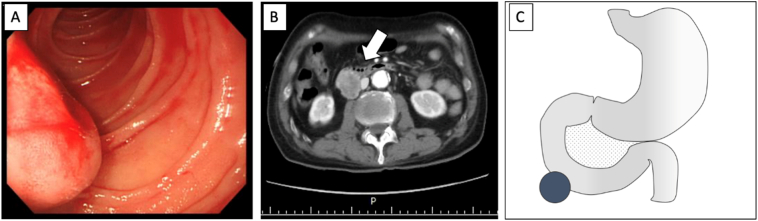
Tumour location and findings in patient one. (A) Endoscopic findings shows bleeding from the apex of the tumour in the 2nd portion of the duodenum. (B) An abdominal enhanced CT scan show a 2.5-cm-sized enhanced tumour between the IVC and the duodenum. Arrow, tumour. (C) Scheme for the site of the tumour. •, tumour. Abbreviations: CT, computed tomography, IVC, inferior vena cava.

**Fig. 2:**
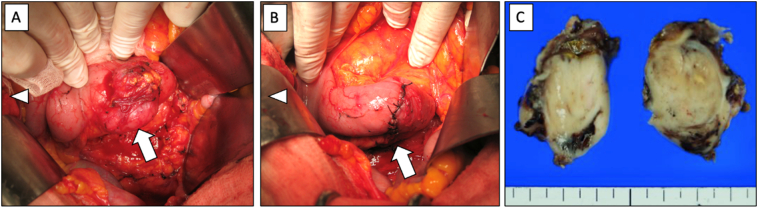
The intraoperative findings in patient one. (A) The tumour on the duodenum did not invade any adjacent organs. Arrow, tumour; Arrowhead, cranial side of the body. (B) We remove the tumour by the wedge resection technique and close the duodenum in a short direction. (C) The cross-section of the removed tumour.

An 84-year-old man sought consultation due to fatigue. Upon clinical examination, the physician found melena. Further blood test result revealed severe anaemia. Hence, he was referred to our hospital. On the same day, he was admitted to our hospital as an emergency case due to gastrointestinal bleeding. The patient had hypertension and received medications. Physical and laboratory examinations are shown in Table 1. Abdominal examination revealed no palpable masses. He was transfused with eight units of red blood cell. An emergency upper gastrointestinal endoscopy was performed, which revealed a 3-cm submucosal tumour located in the 2nd portion of the duodenum (Fig. 1A). Active bleeding was observed from the apex of the tumour. A haemostatic forceps was used to attempt bleeding termination, which was unsuccessful. On the 2nd and 3rd day of admission, repeat endoscopy and haemostatic therapy with transfusion of red blood cells and platelets were performed. The imaging findings are described in Figs. 1B and C. On admission day four, he presented with melena amounting to approximately 300 ml. Endoscopy was insufficient to stop the bleeding, which necessitated emergency surgery. On laparotomy, a 3-cm-sized duodenal submucosal tumour (SMT) was observed in the 2nd portion of the duodenum. No inferior vena cava (IVC) or pancreatic infiltration was observed (Fig. 2A). Wedge resection of the duodenum was performed (Fig. 2B). No postoperative complications were observed. On postoperative day 14, he was discharged.

**Table 1:** Patients' characteristics.

	Patient 1	Patient 2	Patient 3
Physical examination			
Body height (cm)	170	170	160
Body weight (Kg)	58.0	73.0	88.6
Blood pressure (mmHg)	126/51	146/74	133/68
Cardiac rate (beats/min)	102	84	90
			
Laboratory examination			
WBC (×10^3^ /μl)	12.8	5.8	10.2
RBC (×10^4^ /μl)	201	384	474
Hb (g/dl)	6.6	10.1	12.8
Hematocrit (%)	19.7	31.9	38.7
Plt (×10^4^ /μl)	7.1	25.4	20.4
			
Operative record			
Total operative time (min)	147	128	298
Intraoperative blood loss (g)	370	10	1400

Abbreviations: Hb, haemoglobin; Plt, platelets; RBC, red blood cell; WBC, white blood cell.

Macroscopic examination demonstrated that the duodenal tumour appeared as a 35-mm SMT with an ulcer (Fig. 2C). Histopathological examination revealed spindle cell tumour. The tumour showed five mitosis/50 high-power fields (HPFs). Immunohistochemical staining showed that the tumour cells were positive for c-KIT and CD34. He was diagnosed with duodenal GIST. According to Fletcher's risk classification, he was classified as having low–risk GIST. Five years postoperatively, no recurrence was reported.

### 3.2 Case report 2

**Fig. 3:**
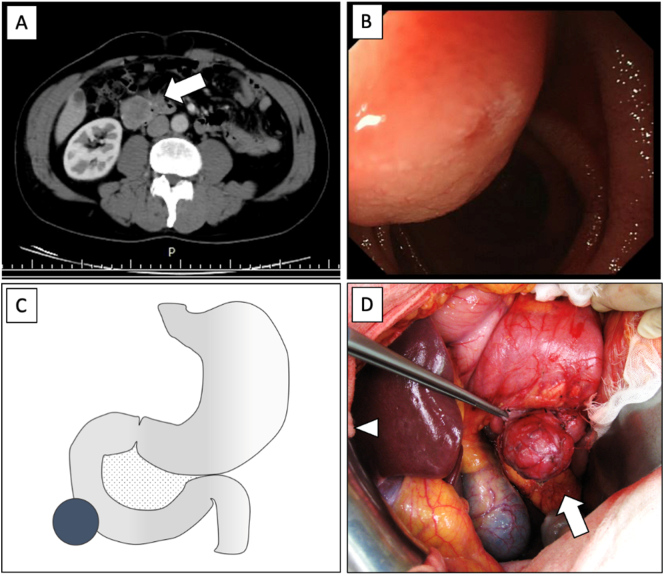
Tumour location and findings in patient two. (A) Abdominal enhanced CT scan shows a 3-cm-sized heterogenous enhanced tumour in the anterior wall of the 2nd portion of the duodenum. Arrow, tumour. (B) Endoscopic findings demonstrate an SMT in the 2nd portion of the duodenum. (C) Scheme for the site of the tumour. •, tumour. (D) No invasion to the adjacent organs is observed. Arrow, tumour; Arrowhead, cranial side of the body. Abbreviation: SMT, submucosal tumour.

A 58-year-old man underwent right inguinal hernia repair at our hospital. However, he developed postoperative anaemia. The patient had a tachyarrhythmia and received medications. Physical and laboratory examinations are shown in Table 1. No mass was palpated. The imaging findings are described in Figs. 3A and B. Endoscopic ultrasound-guided fine-needle aspiration failed to visualise the tumour.

He was hospitalised for elective surgery for SMT. Intraoperatively, a 3-cm extrinsic tumour was observed in the 2nd portion of the duodenum (Fig. 3C, D). No invasion into the adjacent organs was observed. Therefore, wedge resection of the duodenum was performed. No postoperative complications were observed. On postoperative day 19, he was discharged.

Macroscopic examination demonstrated that the 40-mm duodenal tumour resembled an SMT. Histopathological examination revealed spindle cells that had five mitosis/50 HPF and, immunohistochemically, were stained positive for c-KIT and CD34. Consequently, the patient was diagnosed with duodenal GIST. According to Fletcher's risk classification, he was classified as having low–risk GIST. Five years postoperatively, no recurrence was reported.

### 3.3 Case report 3

A 57-year-old woman presented with ischaemic colitis. During consultation, the patient underwent abdominal CT, which incidentally identified a 6-cm-sized retroperitoneal tumour. She declined tumour resection due to diabetes. However, tumour size increased throughout the year. Therefore, she was referred to our department for tumour resection. The patient had a history of myoma uteri.

**Fig. 4:**
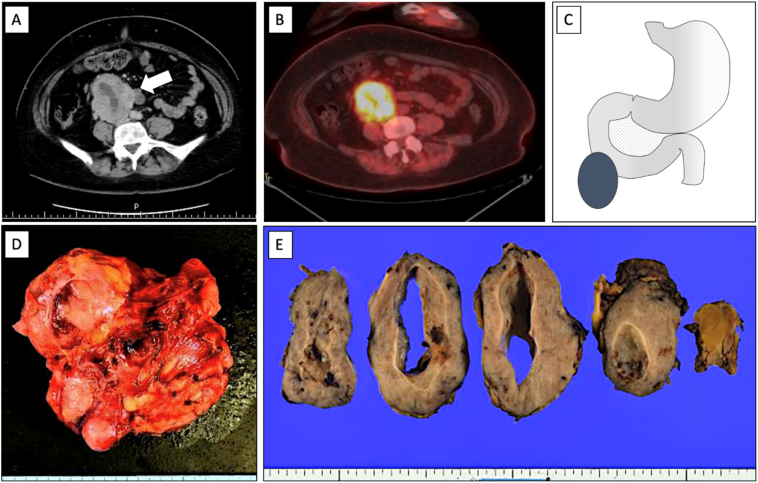
Tumour location and findings in Case 3. (A) Abdominal CT scan demonstrates an 8-cm-sized enhanced tumour next to the 2nd portion of the duodenum, which is consisted of fluid and solid components and presses the IVC (B) PET CT demonstrates that the tumour is uptaken. (C) Scheme for the site of the tumour. •, tumour. (D) Fresh tumour specimen. Macroscopic examination demonstrates that the tumour of size 92 mm was detected as an SMT. (E) The cross-section of the tumour. Abbreviations: CT, computed tomography; PET/CT, positron emission tomography/computed tomography; SMT, submucosal tumour.

Physical and laboratory examinations are shown in Table 1. Abdominal examination revealed a 10-cm palpable mass in the right upper quadrant of the abdomen. The imaging findings are described in Fig. 4A and B. We considered that the tumour was retroperitoneal in origin.

Intraoperatively, a 10-cm-sized giant tumour was located adjacent to the inferior duodenal flexure (Fig. 4C). The tumour was thought to originate from the duodenum as an SMT. IVC or pancreatic infiltration was not observed. Wedge resection of the duodenum was possible, despite the large size of the tumour. However, simple closure of duodenal wall was not possible due to the broad range of the wall. Therefore, a side-to-side anastomosis with the jejunum was performed. No postoperative complications were observed. On postoperative day 21, she was discharged.

**Fig. 5:**
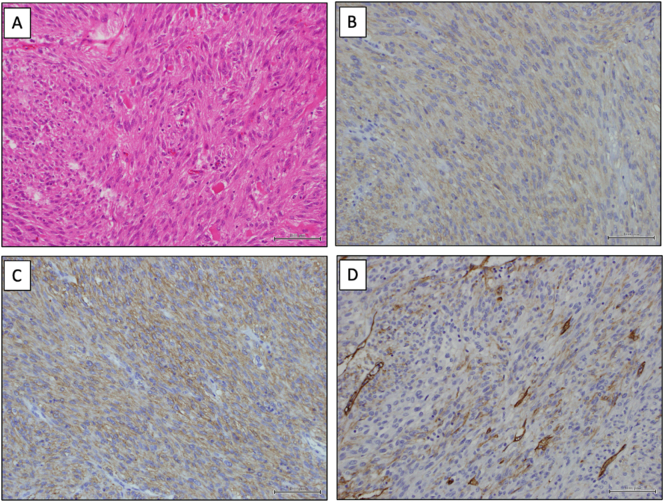
Histopathological examination in patient three. (A) Haematoxylin and eosin staining of the tumour reveals spindle cells. The tumour has 1 mitosis/50 HPF. (B) Immunohistochemical staining for c-KIT shows positive cells. (C) Immunohistochemical staining for CD34 shows positive cells. (D) Immunohistochemical staining for DOG1 shows positive cells. Scale bar, 100 μm.Abbreviation, HPF, high-power field.

The macro- and microscopic findings are shown in Fig. 4, Fig. 5, respectively. Consequently, she was diagnosed with duodenal GIST (Fig. 5). According to Fletcher's risk classification, she was classified as having high–risk GIST, which necessitated adjuvant imatinib at a dose of 400 mg/day orally. Three years postoperatively, she experienced no recurrence.

## 4 Discussion

Herein, three patients with GISTs in the 2nd portion of the duodenum underwent wedge resection. Pancreatoduodenectomy was avoided in all patients. Recurrence was not noted in any patient. This report would help all clinicians identify clinical features and treatments of duodenal GISTs.

Previous reports have summarised the clinicopathological features of duodenal GISTs [5], [6], which most frequently occur in the 2nd, followed by the 3rd portion of the duodenum. All patients in our report presented with GIST in the 2nd portion of the duodenum, which is adjacent to the 3rd portion.

GIST commonly manifests most commonly with bleeding, followed by epigastric pain, jaundice, and bowel obstruction [7]. Compared with gastric GISTs, duodenal GISTs have higher incidence of bleeding [7]. Herein, the first patient presented with bleeding, which necessitated haemostatic therapy providing temporary bleeding relief. Upon recurrence, emergency laparotomy and wedge resection were performed because endoscopic haemostasis was unsuccessful.

Regarding the surgical procedure, local resection with negative margins is sufficient because GISTs are not associated with lymph node metastasis [8]. However, pancreatoduodenectomy or pylorus-preserving pancreatoduodenectomy for tumours close to the ampulla of Vater is required [3]. In contrast, segmental duodenectomy should be performed on large-sized tumours that do not preserve duodenal wall during local resection. This study suggested that local resection was sufficient for managing patients with duodenal GISTs without recurrence. Lee et al. indicated that limited resection including wedge resection of the duodenum was performed in 62% of 118 patients with duodenal GISTs [5]. They also suggested that limited resection was feasible and effective procedure in patients with especially small-sized and antimesenteric-sided duodenal GISTs regarding complications.

The Japanese clinical practice guidelines for GIST recommend resection of tumours measuring 2.5 cm because it is easy to perform [9]. However, the guidelines do not recommend laparoscopic resection of malignant GISTs. However, the minimally invasive nature of laparoscopic resection has led to its increased use for gastric GISTs [10]. A recent study that evaluated long-term outcomes of laparoscopic resection has found that it was feasible to perform in GISTs measuring >5 cm [11]. In 2008, Hiki et al. developed the laparoscopy endoscopy cooperative surgery (LECS), a minimally invasive surgery, for management of gastric SMTs and GISTs [12]. Recently, the LECS procedure successfully and safely managed patients with duodenal tumours measuring <2 cm [13]. Laparoscopic wedge resection or LECS are potentially feasible and safe surgical procedures for duodenal GISTs. However, it is more important to consider the type of oncological resection to be performed compared with the type of minimally invasive surgery. Therefore, neoadjuvant chemotherapy may be helpful for large tumours or tumours suspected of invading adjacent organs. Specifically, neoadjuvant imatinib treatment can decrease tumour size and prevent extensive resection and tumour rupture [14]. Huang et al. found that tumours in the 3rd portion of the duodenum were larger and had more severe surgical complications compared with other portions of the duodenum in patients with resected GIST from the duodenum and proximal jejunum [15]. Pathological diagnosis of GIST is required when administering neoadjuvant chemotherapy for large GISTs. Endoscopic ultrasonography (EUS) and EUS-guided biopsy are helpful in diagnosing GIST and evaluating neoadjuvant therapy [16]. Herein, EUS was not performed in patient one due to bleeding. However, EUS and EUS-guided-fine-needle aspiration were attempted in patients two and three. However, it was unsuccessful in detecting the tumour.

Regarding recurrence risk stratification, Fletcher et al. [17], Miettinen and Lasota [18], and Joensuu et al. [19] classifications are generally used. Fletcher's risk stratification is a well-established method for recurrence risk classification. Miettinen et al. established risk stratification by adding the site of organ affected by the tumour, to its size and mitosis count [18]. In addition, the risk stratification method of Joensuu et al. added tumour rupture as a variable [19]. Compared with GISTs in other organs, duodenal GISTs have poorer prognosis.

Recurrence rate of duodenal GISTs was higher compared with gastric GISTs [7]. Adjuvant chemotherapy with imatinib for patients with high-risk GISTs prevents recurrence [20]. In our report, patient three was classified as having high-risk GIST, which prompted adjuvant imatinib. Currently, the patient has not experienced recurrence three years postoperatively.

## 5 Conclusion

We describe three patients with duodenal GIST in whom duodenal wedge resection was performed. Our findings suggest the potential use of this resection in managing tumours not adjacent to the ampulla of Vater. In the future, we should adopt less invasive procedures for duodenal GISTs to avoid complications and improve postoperative quality of life. Moreover, giant localised duodenal GISTs may be resected by wedge resection after neoadjuvant treatment. However, future studies with larger sample size are required to validate our suggestion.

## CRediT authorship contribution statement

Tomoaki Ito: Writing the manuscript.

Tomoyuki Kushida, Mutsumi Sakurada and Kenichiro Tanaka: Making the figures.

Koichi Sato: Supervision.

Hiroshi Maekawa: Supervision.

## Provenance and peer review

Not commissioned, externally peer-reviewed

## Funding

We have no source of funding to declare.

## Ethical approval

Not applicable

## Consent

Written informed consent was obtained from the patient for publication of this case report and accompanying images. A copy of the written consent is available for review by the Editor-in-Chief of this journal on request.

## Registration of research studies

In accordance with the Declaration of Helsinki 2013, the Editors of IJS Case Reports require that reports that are ‘First in Man’ studies should be registered prospectively and failing that retrospectively. There are many places to register your First in Man case report:

• Clinicaltrials.gov – for all human studies – free

• Chinese Clinical Trial Registry chictr.org.cn – for all human studies – free

• Researchregistry.com – for all human studies – charge

• ISRCTN.com – for all human studies – charge

• There are many national registries approved by the UN that can be found here

Elsevier does not support or endorse any registry.

## Guarantor

The Guarantor is the one or more people who accept full responsibility for the work and/or the conduct of the study, had access to the data, and controlled the decision to publish

## Declaration of competing interest

We have no declaration of interest.
